# A mixed-method process evaluation of guided online cognitive behavioral therapy for insomnia in patients with borderline personality disorder

**DOI:** 10.1186/s12888-026-07859-8

**Published:** 2026-02-12

**Authors:** Shanna van Trigt, Femke van Nassau, Tanja van der Zweerde, Eus J. W. van Someren, Annemieke van Straten, Hein J. F. van Marle

**Affiliations:** 1 Amsterdam UMC, Location Vrije Universiteit, Psychiatry, De Boelelaan 1117, Amsterdam, the Netherlands; 2 Amsterdam Public Health Research Institute, Mental Health Program, Amsterdam, the Netherlands; 3 Vrije Universiteit Amsterdam, Clinical, Neuro and Developmental Psychology, Amsterdam, the Netherlands; 4 Department of Public and Occupational Health, Amsterdam Public Health Research Institute, Amsterdam UMC, Location Vrije Universiteit, Amsterdam, the Netherlands; 5 Wagner Psychologists, Utrecht, the Netherlands; 6 GGZ inGeest Mental Health Care, Amsterdam, The Netherlands; 7 Amsterdam Neuroscience, Mood, Anxiety, Psychosis, Sleep & Stress Program, Amsterdam, the Netherlands; 8 Department of Sleep and Cognition, Netherlands Institute for Neuroscience, an Institute of the Royal Netherlands Academy of Arts and Sciences, Amsterdam, the Netherlands; 9 Department of Integrative Neurophysiology, Center for Neurogenomics and Cognitive Research, Amsterdam Neuroscience, Vrije Universiteit, Amsterdam, the Netherlands; 10 ARQ National Psychotrauma Center, Diemen, the Netherlands

**Keywords:** Borderline personality disorder, Cognitive behavioral therapy for insomnia, Insomnia, Sleep, Process evaluation, Implementation

## Abstract

**Background:**

Borderline personality disorder (BPD) is often associated with insomnia, which may contribute to a vicious cycle of reciprocal exacerbation of sleep disturbance and BPD symptoms. Online cognitive behavioral therapy for insomnia (iCBT-I) has shown promise for treating insomnia in BPD patients. This study evaluated the key determinants, underlying processes, and change mechanisms of successful implementation of guided iCBT-I in BPD patients.

**Methods:**

In the context of our clinical trial, we conducted this mixed-method process evaluation in 30 patients with BPD and comorbid insomnia complaints (mean age 29.5 years ± 8.72) that received guided iCBT-I. Our process evaluation was guided by four dimensions of the theoretical RE-AIM framework: Reach (e.g., reason participation), Effectiveness (e.g., perceived symptom reduction), Implementation (e.g., treatment delivery), Maintenance (e.g., sustained adherence). We combined insights from quantitative patient-reported evaluation of treatment satisfaction and adherence and therapist log data, with qualitative exploration of patient experiences through in-depth interviews in a subsample of five patients.

**Results:**

21 patients (70%) completed the intervention. Patients appreciated the online modality and emphasized the importance of responsive and personalized (videocall) therapist guidance and active reminders. Patients found behavioral strategies, such as fixed bedtimes and an evening winding-down routine particularly helpful, while more introspective and cognitive techniques were often perceived as challenging. Qualitative effectiveness evaluations aligned with clinical trial outcomes on BPD, insomnia and arousal-related symptoms. Finally, patients expressed a strong desire for more and well-integrated sleep treatment into regular BPD care trajectories.

**Conclusions:**

Guided iCBT-I proved a feasible and promising intervention for patients with BPD, with successful implementation contingent upon personalized guidance, focus on behavioral strategies tailored to individual needs, and solid integration into BPD care.

**Supplementary Information:**

The online version contains supplementary material available at 10.1186/s12888-026-07859-8.

## Introduction

Borderline personality disorder (BPD) is a severe psychiatric disorder characterized by profound emotion dysregulation, affecting 1–5% of the general population and 11–35% of the clinical population [1]. Given the high rates of suicide, comorbidities, use of mental health care, and associated functional impairment, BPD constitutes a high personal and societal burden [2].

A growing body of literature highlights the essential role of sleep in emotion regulation [3, 4]. Sleep disturbances such as insomnia impair overnight alleviation of emotional distress [4], thereby aggravating daytime emotion dysregulation. Based on these insights, insomnia is increasingly considered a causal factor and important treatment target in psychiatric disorders [5]. This may hold especially for BPD patients, given the central role of emotion dysregulation in the disorder and high prevalence of comorbid insomnia and other sleep disturbances [6, 7], which likely create a self-perpetuating cycle of reciprocal exacerbation of disturbed sleep and BPD symptoms [8]. Insomnia symptoms in BPD have been shown to aggravate affective instability and risk of self-harm and suicidal behavior [9, 10], and may hamper BPD treatment effectiveness [11].

Cognitive behavioral therapy for insomnia (CBT-I) and its online versions (iCBT-I) are the preferred treatment for insomnia [12]. Meta-analytic evidence indicated that (i)CBT-I effectively treats insomnia and psychiatric symptoms in some psychiatric populations (e.g., depression, PTSD) [13]. However, for certain psychiatric populations (e.g. psychiatric inpatients or patients with psychotic and bipolar disorders), the benefit of (i)CBT-I on psychiatric symptoms is less consistent and studies in these populations remain limited [13, 14]. Our recent randomized controlled trial (RCT) was the first to investigate the effectiveness of guided iCBT-I (compared to keeping a sleep diary only) in patients with BPD (traits) and comorbid insomnia [15]. Conducted within Dutch mental healthcare settings, the RCT investigated iCBT-I administered prior to or in the early stages of specialized BPD treatment. Results indicated that iCBT-I effectively alleviated insomnia and some arousal-related BPD symptoms (i.e., anger attacks and morning tension), while it did not significantly reduce overall BPD severity or other mental health-related outcomes like depression or quality of life [16].

So far, the clinical implementation of (i)CBT-I in psychiatric settings is very limited, partly due to the lack of familiarity with and training in CBT-I among mental healthcare professionals, and insomnia remains treated mainly pharmacologically [13, 17, 18]. Efforts to improve the implementation of (i)CBT-I are therefore needed in addition to studying its clinical effectiveness. Intervention process evaluations, which may address both organizational contexts (e.g., provider-related barriers) and patient perspectives (e.g., individual patient needs and preferences), facilitate insight into determinants of treatment effectiveness and successful implementation [19]. A recent process evaluation of guided iCBT-I in individuals with anxiety- and trauma-related mental health complaints offered important recommendations for implementation, such as the need for individualized modifications of content and guidance to cater to diverse individual needs [20]. Evaluation of these processes is highly recommended for pragmatic clinical trials and the implementation of novel treatment strategies to help translate research findings into clinical practice [21]. Given that iCBT-I is a new intervention strategy for BPD and digital health research is still a relatively new field within this population, understanding how iCBT-I is received by patients with BPD is important for informing future implementation efforts. As part of our RCT on the effectiveness of iCBT-I for BPD, we therefore conducted a process evaluation in patients assigned to the iCBT-I condition. In this mixed-method process evaluation, we aimed to understand the key determinants and underlying processes and mechanisms of successful implementation from the patient perspective, for example regarding delivery methods, (non-)effective treatment components, and contextual influences. By combining in-depth qualitative exploration of patient experiences with quantitative patient- and therapist-reported insights, we sought to guide future implementation of iCBT-I for patients with BPD in clinical practice.

## Methods

### Design

This mixed-method process evaluation was conducted in parallel with our RCT, which evaluated the effectiveness of guided iCBT-I, compared to a sleep diary control condition, prior to or during the early stage of standard BPD treatment. The process evaluation used a convergent triangulation design, in which we collected quantitative and qualitative data simultaneously, analyzed the data separately, and merged the data at the point of interpretation. The study was theoretically guided by the RE-AIM framework, which evaluates the impact of health promoting interventions in real-life settings [22] (i.e., Reach, Effectiveness, Adoption, Implementation and adherence, Maintenance of treatment effects). Since we focused on patient perspectives, we only used the following dimensions in our process evaluation: Reach (e.g., reasons for participation), Effectiveness (e.g., perceived symptom reduction), Implementation (e.g., evaluation of intervention components), and Maintenance (e.g., sustained use of techniques) and reported our results along this structure. The study was approved by the VU Medical Centre Medical Ethics Committee (NL76232.029.20) as part of the RCT [15] and registered with the Netherlands Trial Register (NL9776, date: 4-10-2021), which was recently discontinued and incorporated into the national registry Overview of Medical Research in the Netherlands (OMON) (registration number: NL-OMON21458).

### Population

Patients in this process evaluation were participants of the RCT randomized to the iCBT-I condition. Recruitment was conducted at three specialized mental health care centers in the Netherlands. Inclusion criteria of the RCT were: DSM-5 BPD diagnosis, or other personality disorder (PD) diagnosis with ≥ 4 BPD traits; 18–65 years; insomnia complaints (Insomnia Severity Index (ISI) score ≥ 10 [23]); able to complete online questionnaires and diaries in Dutch. Exclusion criteria were: current bipolar or psychotic disorder diagnosis, assessed by self-report; severe alcohol or drug dependency; having received (i)CBT-I in the past 3 months.

### Procedure

**Fig. 1:**
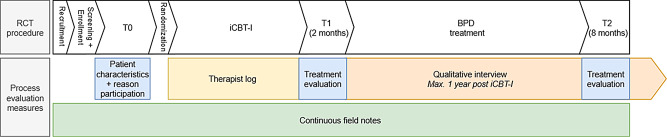
Timing of the process evaluation procedure in parallel to the randomized controlled trial (RCT). BPD: borderline personality disorder, iCBT-I: online cognitive behavioral therapy for insomnia

We recruited patients at intake or on the waitlist for BPD treatment. Mental healthcare providers asked patients for permission to be contacted by the researchers, who then provided study information to the patient. After providing informed consent and eligibility screening, patients enrolled in the study. Assessments were conducted at baseline (T0), prior to randomization to either iCBT-I or the control condition, and repeated at 2-month post-iCBT-I follow-up (T1) and 8-month follow-up, after subsequent BPD treatment (T2). Figure 1 displays the procedure of the process evaluation assessments in parallel to the RCT.

The quantitative questionnaires were collected online at T0, T1 and T2 in all patients of the RCT assigned to the iCBT-I condition. For these participants, a therapist log was kept during the iCBT-I treatment and researcher field notes were made throughout the entire trial duration. Regarding qualitative data, we expanded the original study protocol after the RCT had commenced to include the in-depth evaluation of patient experiences by individual semi-structured interviews. After this, we invited all patients allocated to the iCBT-I condition (regardless of intervention completion or discontinuation) to participate in the interviews after T1, for which they needed to provide separate informed consent. We aimed to recruit participants representing different phases of treatment completion also containing views on longer-term satisfaction and effectiveness. To minimize the risk of recall bias, we did not invite patients who had completed the intervention more than 1 year ago. Interviews were conducted via videocall by a research assistant (not involved in the iCBT-I guidance or data collection of the RCT), tape recorded, manually transcribed verbatim, and pseudonymized. Recordings were securely stored with access restricted to authorized researchers and deleted after final data-analyses. We aimed to collect data until thematic data saturation had been reached. Data was considered saturated when no new themes were introduced in three consecutive interviews.

### Intervention

The RCT evaluated adding iCBT-I prior to or in the early stages of standard BPD treatment. Patients were preferably included at least 2 months before starting their awaited BPD treatment, while allowing a partly overlap in iCBT-I and early stages of BPD treatment.

The guided iCBT-I intervention i-Sleep consists of five sessions [24, 25]. Detailed descriptions of session content and the specific treatment components that were evaluated in this process evaluation can be found in Table 1. We used the mental health version of i-Sleep, in which case vignettes are adapted to represent patients with comorbid psychiatric symptoms. The intervention lasted 5–8 weeks and instructed patients to complete one session per week (with room to postpone a session for 3 additional weeks in case more time to complete a session or implement specific components was needed) next to filling out a daily sleep diary during the entire treatment period. Patients received written online feedback after each session, reminders to complete a session (online and by phone, according to a standardized procedure), and three videocall appointments of maximum 30 min for additional and more personal guidance (at the start, after 2 weeks, and after 4 weeks). Typically, i-Sleep does not include videocall guidance. This was deemed necessary after treating the first patients, given the high disease burden of BPD, and added in the early stage of the RCT to enhance motivation, adherence, and continuation. Videocall guidance was therefore offered to 24 of the 30 patients. The intervention was delivered via the web-based platform of the Netherlands Sleep Registry (www.slaapregister.nl) and a mobile sleep diary-application, which was linked to the web-based platform allowing patients to review their personal sleep diary data. Guidance was delivered by one therapist (main researcher, SvT), a doctoral researcher in psychiatry with a background in clinical orthopedagogy, who was trained by a psychologist (TvdZ) with expertise in iCBT-I and i-Sleep and participated in weekly supervision meetings with other i-Sleep therapists that provided guidance in parallel clinical trials [26].

**Table 1:** Content of the five i-Sleep sessions and specific intervention components

Session	Content session	Evaluated components
1	Psychoeducation on (disturbed) sleep and the importance of good sleep hygiene. The session covers factors that influence sleep, such as physical exercise, caffeine, nicotine, and alcohol intake, and optimal bedroom surroundings.	- **Lifestyle adjustments**: e.g., exercise, relaxation throughout the day, caffeine/alcohol/drug consumption - **Bedroom surroundings**: e.g., temperature, light, sound - **Evening ritual**: e.g., relaxing activities before bed to unwind
2	Introduces sleep restriction therapy: restrict the time in bed to the average sleep duration (minimum of 5 h) if sleep efficiency (i.e., % of time slept relative to time spent in bed) was below 80%. Bedtimes could be gradually extended by 15 min if sleep efficiency was above 85% for a week. Patients were advised not to deviate from bedtimes on weekend-nights and to avoid daytime sleeping (or at least restrict to naps of less than 30 min, no later than 3pm). As part of stimulus control patients were also advised not to go to bed before feeling sleepy, use the bedroom for sleep and sex only, and get up at night when lying awake for longer than 15–30 min, only to return when feeling sleepy again.	- **Fixed bedtimes**: maintaining consistent sleep- and wake times - **Sleep restriction**
3	Covers relaxation exercises and techniques to reduce intrusive thoughts and rumination.	- **Relaxation**: e.g., audio-recorded muscle relaxation exercises - **Rumination**: e.g., a daily 15-min window to write down thoughts and worries, thought blocking techniques, trying to stay awake at night
4	Covers cognitive techniques to tackle dysfunctional cognitions about sleep. The most common dysfunctional misconceptions about insomnia and its consequences are discussed, and strategies to challenge and change these cognitions are introduced.	- **Sleep-related cognitions**: e.g., reappraisal, reattribution, attention shifting, hypothesis testing
5	Summary of the content of the four previous sessions, evaluation of progress, and patients were instructed to develop a plan to maintain and further improve their sleep in the short- and longer-term future.	- **Future plan**

### Standard BPD treatment

Patients received the iCBT-I intervention prior to or in the early stages of standard BPD treatment within Dutch mental healthcare settings. This standard care consisted of evidence-based psychotherapies as recommended by national guidelines, including Dialectical Behavior Therapy (DBT), Schema Therapy, Mentalization-Based Therapy (MBT), Systems Training for Emotional Predictability and Problem Solving (STEPPS), and guideline-informed psychiatric care. Patients were included after intake, preferably at least 2 months before starting their awaited BPD treatment, while allowing a partly overlap in iCBT-I and early stages of BPD treatment to prioritize timely access to care.

### Measures

#### Quantitative assessments

Details regarding the assessment of clinical baseline characteristics can be found in the appendix.

##### Questionnaires

Patient characteristics, reasons to participate in the RCT, and history of followed sleep treatments were assessed at baseline (T0). Treatment evaluation was assessed at T1 and T2. At T1, patients were asked to rate their satisfaction with i-Sleep and the online guidance on a scale of 1 (not at all satisfied) to 10 (very satisfied), and how helpful the different components of i-Sleep (Table 1) have been for improving their sleep (1 = not at all effective, 10 = very effective). Patients were asked for suggestions on how to improve i-Sleep or its guidance in an open-text field. At T2, patients rated to what extent they carried out the i-Sleep components after completion of the treatment (1 = never performed again, 10 = performed daily). The complete questionnaires can be found in the appendix.

##### Therapist log and field notes

The web-based platform logged the completed sleep diaries and i-Sleep sessions. Therapist logs additionally indicated duration of the videocall appointments and time spent on written feedback, as well as the number of postponed sessions and reminders needed. Continuous researcher field notes during the entire trial period indicate reasons for not participating in the RCT, for dropping out of the RCT, and for discontinuing i-Sleep.

#### Qualitative assessments

We conducted semi-structured interviews using a topic guide based on the RE-AIM framework (provided in the appendix) to qualitatively evaluate BPD patient experiences with i-Sleep. The topic guide contained: (1) reasons for participation, goals, and expectations; (2) use of and experiences with the intervention (components); (3) digital method; (4) therapeutic relationship and guidance; (5) perceived effectiveness (on insomnia and BPD complaints); (6) combination and timing of iCBT-I with subsequent BPD treatment; (7) continued use of intervention (components). A complete overview of all process evaluation objectives with their respective measures for each of the adopted RE-AIM dimension is provided in Table 2.

**Table 2:** Objectives and their respective measures structured by four of the five RE-AIM dimensions: Reach, Effectiveness, Implementation, and maintenance

	BPD patients		Therapist log	Field notes
	Quest. (total sample)	Interview (subsample)		
***Timepoint***	*T0/T1/T2*	*< 1 year post-iCBT-I*	*Continuously during iCBT-I*	*Continuously entire trial period*
**Reach of patients**				
Recruitment procedures				●
Reasons for starting and (dis)continuing trial participation/iCBT-I	T0	●		●
Patient characteristics	T0			
**Effectiveness**				
Patients’ perceived effectiveness	T1	●		
Patients’ satisfaction, views, and experiences with iCBT-I	T1	●		
**Implementation**				
Treatment uptake and patient engagement (e.g., completed sessions, reminders needed)		●	●	
Provided therapist guidance (e.g., time spent on feedback/videocalls)			●	
Patients’ views on specific iCBT-I components	T1	●		
Patients’ views on digital implementation		●		
Patients’ views on therapeutic guidance		●		
Patients’ views on combining iCBT-I with standard BPD treatment		●		
**Maintenance**				
Patients’ long-term adherence	T2	●		
Patients’ intentions to use iCBT-I in the future		●		
**Overall**				
Recommendations for improvement of iCBT-I and its implementation	T1	●		

### Analyses

Quantitative data were prepared and analyzed descriptively using R (version 4.4.3) [27]. Descriptives of demographic and clinical characteristics, and questionnaire, log, and field note data were displayed as mean ± standard deviation (SD) or proportionally.

Qualitative data were analyzed using a codebook thematic analysis approach, which is recommended for applied (implementation) health research, and offers flexibility in using both inductive and deductive analyses, allowing integration of the theoretical RE-AIM framework while remaining open to generating other ideas [28]. MAXQDA 2022 [29] was used for coding the interview transcripts. Codes were assigned to label our understanding of the meaning of data segments deemed relevant for the research question. After coding the first three interviews, two researchers (SvT and FvN) discussed the initial interpretations and codes to establish a codebook, which was sent to other researchers (AvS and HvM) for feedback. The codebook provided an organized framework based on our research question and the RE-AIM topics. Next, all interviews were coded based on the established codebook. While the total of 5 included interviews did not allow us to meet the predefined criterion for data saturation, no new themes emerged in the subsequent 2 interviews after establishing the codebook based on the first 3 interviews, suggesting that the core themes were well-captured within this sample. All coded segments were clustered into themes that explore potential patterns of shared meaning and core concepts, which were reviewed (checked if themes are coherent, distinct, and adequately capture all data) and clearly named. The codebook is provided in the appendix.

Subsequently, a mixed-method process evaluation report was created by triangulating qualitative and quantitative data, evaluating any contradictions or confirmations between the findings from both methods. Relevant and illustrative quotes from interviewed participants were selected after discussion among the research team. Additions to clarify quotes were presented between brackets.

## Results

In total, 30 patients were allocated to the iCBT-I intervention condition. All patients completed the baseline questionnaire. The treatment evaluation questionnaire was completed by 19 (63%) patients at T1 and by 21 (70%) patients at T2. Of the 30 patients allocated to receive iCBT-I, 13 (43%) patients were invited after inclusion of the qualitative interviews in the study, of which, 5 patients (38%) participated in the interviews. Interviews had a mean duration of 39 min, ranging from 32 to 50 min. Therapist log data was largely complete with *n* = 57 video call durations, *n* = 101 written feedback durations, *n* = 482 logs for reminders, and *n* = 123 logs for postponement of sessions. Information was missing for the duration of one videocall, and for written feedback of 7 sessions divided over 4 patients.

### Reach

#### Patient characteristics and reasons for participation

The mean age of the total sample was 29.5 years ± 8.72 with 70% females. Average baseline severity of BPD and insomnia both indicated clinical symptom severity and appeared comparable in the total sample (*n* = 30) and interview subsample (*n* = 5) (see Table 3 for all demographic and clinical sample characteristics). Almost all patients (97%) reported they had never received (i)CBT-I or other psychotherapeutic sleep treatments in the past. The primary motivation for patients to participate was to address their insomnia symptoms (83%), which was also confirmed in the interviews: all patients expressed the desire to improve often long-standing sleep problems and learn practical tools to improve sleep. Patients often expressed more than one reason for participating. Improving daytime functioning or quality of life was also a common reason (70%). Beyond personal health reasons, 53% was motivated to contribute to scientific research, which resonated with reasons mentioned in part of the interviews. While all patients were recruited through their mental healthcare provider, only 23% reported the advice of a healthcare professional as reason for participation.

**Table 3:** Demographic and clinical baseline characteristics

	Interview subsample (*n* = 5)	Total sample (*n* = 30)
**Demographic characteristics**		
Sex assigned at birth: female, *n* (%)	2 (40%)	21 (70%)
Age in years, M (*SD*)	35.0 (11.4)	29.5 (8.72)
Country of birth, *n* (%)		
Netherlands	4 (80%)	24 (80%)
Belgium	0 (0%)	1 (3.3%)
Caribbean	1 (20%)	1 (3.3%)
South America	0 (0%)	4 (13.3%)
Educational status, *n* (%)		
Primary or no education	0 (0%)	2 (6.7%)
Secondary education	5 (100%)	18 (60%)
Tertiary education	0 (0%)	10 (33.3%)
Occupational status, *n* (%)		
Employed full-time	5 (100%)	16 (53.3%)
Employed part-time	0 (0%)	6 (20%)
Social assistance / incapacity	0 (0%)	5 (16.7%)
Unemployed	0 (0%)	1 (3.3%)
Studying	0 (0%)	2 (6.7%)
**Clinical characteristics**		
BPD symptom severity (BPDSI), M (*SD*)	29.9 (*8.22*)	27.7 (*10.5*)
Amount of BPD traits (SCID-5-P), M (*SD*)	4.80 (*0.84*)	5.53 (*1.11*)
Personality disorders (SCID-5-P), *n* (%)		
Borderline PD	3 (60%)	25 (83.3%)
Avoidant PD	2 (40%)	5 (16.7%)
Obsessive-compulsive PD	0 (0%)	1 (3.3%)
Paranoid PD	1 (20%)	1 (3.3%)
Other PDs ^a^	0	0
Other psychiatric comorbidities (MINI), *n* (%)		
Major Depressive Episode	2 (40%)	11 (36.7%)
Social Anxiety Disorder	2 (40%)	7 (23.3%)
Generalized Anxiety Disorder	3 (60%)	14 (46.7%)
Panic Disorder	0 (0%)	3 (10%)
Post-Traumatic Stress Disorder	3 (60%)	17 (56.7%)
Insomnia symptom severity (ISI), M (*SD*)	17.2 (*5.72*)	17.4 (*4.54*)
Self-reported sleep/wake disorders other than insomnia, *n* (%)		
Narcolepsy	1 (20%)	4 (13.3%)
Sleep apnea	0 (0%)	7 (23.3%)
Circadian rhythm sleep disorder	1 (20%)	6 (20%)
Restless legs/periodic limb movement disorder	1 (20%)	4 (13.3%)
Parasomnia	0 (0%)	3 (10%)
Missing	0 (0%)	1 (3.3%)
Psychotropic medication use, *n* (%)	2 (40%)	13 (43.3%)

Note. BPDSI: Borderline Personality Disorder Severity Index-5 4-week version, ISI: Insomnia Severity Index, MINI: Mini-International Neuropsychiatric Interview, SCID-5-P: Structured Clinical Interview for DSM-5 Personality Disorders. ^a^ Other personality disorders (PDs; i.e. Dependent PD, Schizotypal PD, Schizoid PD, Histrionic PD, Narcissistic PD, Antisocial PD) did not occur in the sample

#### Discontinuation of iCBT-I

The rate of attrition was 30%, as 21 patients (70%) completed the iCBT-I intervention (4–5 sessions), 5 (17%) partly completed iCBT-I (2–3 sessions), and 4 (13%) did not complete iCBT-I (0–1 sessions). Of the patients that discontinued iCBT-I, three chose to discontinue iCBT-I while continuing participation in the RCT, explaining that insomnia was not their most prominent complaint rendering the perceived burden of the treatment disproportionate to the expected benefit. The remaining six patients dropped out of the entire RCT, of which three formally quit as they perceived the burden of participation too high next to their BPD-related difficulties, and three were lost to follow-up.

### Effectiveness

During the interviews, patients indicated that overall, they did not experience a clear direct effect of iCBT-I on their BPD symptoms, although most found it difficult to differentiate effects of iCBT-I and BPD treatment. While their BPD symptoms were primarily addressed by BPD treatment, improvements in sleep may have been a contributing factor for some: *“I think that my symptoms were alleviated more by the BPD treatment*,* but that subconsciously*,* my sleep played a very big role in it.”* – Patient 1 (male, 25 years). Although improvements in sleep may not be sufficient to reduce overall BPD severity or require more time, some patients did experience a reduction in specific BPD symptoms due to improved sleep, such as restlessness, irritability and worrying: *“I think where it may have helped*,* i-Sleep*,* is that when I sleep better*,* I’m a little less irritable during the day*,* but it didn’t solve my borderline completely*,* no*,* unfortunately not. I think that just takes time.”* – Patient 4 (female, 22 years). All interviewed patients did notice a positive effect of iCBT-I on their sleep. They primarily mentioned falling asleep faster and sleeping through the night, which they attributed to feeling calmer, experiencing less tension at night, and building up more sleep pressure from adjusting their bedtimes and sticking to a more consistent sleep schedule. One patient also struggled with early morning awakenings and noted improvements in this area as well. Most patients moreover mentioned feeling less tired during the day and having less worries and negative thoughts concerning sleep. For some, iCBT-I also positively impacted on their daily lives. Specifically, having more routine and a consistent sleep-wake schedule led to more regular eating habits and cooking more often. While one patient experienced strong daytime fatigue during the initial phase of sleep restriction, no participants reported any negative long-term effects on their sleep.

### Implementation

Based on log data, on average, sessions were postponed by 1.93 weeks (± 1.28), leading to a mean duration of 6.93 weeks to complete the iCBT-I intervention. The average number of completed sessions was 3.70 (± 1.68) in the total sample and 4.80 (± 0.45) in the interview subsample. Patients completed 28.9 sleep diaries (± 13.6) on average during the intervention period. The videocall appointments were offered to 24 patients (80%), as these were added to the iCBT-I guidance in a later stage of the trial. Of these patients, 23 (96%) completed the first videocall at the start of the intervention (mean duration 12 min ± 3.61), 19 (79%) completed the second appointment (mean duration 26 min ± 5.75), and 16 (67%) completed the third (mean duration 26 min ± 5.54). The mean time spent by the therapist on providing the written feedback was 21 min per session (± 7.74). Together with the videocall appointments, the average total time spent on iCBT-I guidance was 107 min (± 53.8) per patient. When only considering the patients that completed the intervention, this was 137 min (± 30.3).

The treatment evaluation questionnaire demonstrated the overall patient-reported satisfaction with the iCBT-I intervention was, on average, rated a 5.79 (± 1.40) out of 10 (Table 4). Notably, satisfaction among patients that did not complete the intervention (*n* = 2) was considerably lower, which both rated the overall satisfaction a 4. Among patients who completed iCBT-I (*n* = 17), overall satisfaction was M = 6.00 ± 1.31.

#### Perceived benefit of specific iCBT-I components

##### Sleep restriction and fixed bedtimes

Most patients mentioned that the core principles of sleep restriction, which involved reduction of time spent in bed and maintaining consistent bedtimes, were the most effective for improving their insomnia symptoms. For some, their concrete, behavioral nature was perceived as more helpful than abstract or cognitive components: *“Actually doing something*,* where you see an immediate effect*,* worked better for me than more vague stuff; rationalizing or positive self-talk*,* which don’t really help me. With sleep restriction I immediately noticed I felt more tired*,* I slept longer*,* and I also slept more soundly. I honestly thought: ‘This is very good’.” –* Patient 3 (female, 34 years). Sticking to fixed bedtimes was particularly considered beneficial, which was also reflected in the treatment evaluation questionnaire (M = 7.00 ± 1.79) (Table 4). Sleep restriction helped to impose fixed bedtimes and much-needed routine: *“Yes*,* that [sleep restriction] positively surprised me. I often went to bed very early and then woke up in the middle of the night. It was really hard; I would wake up around 3:00 a.m. and just kept tossing and turning*,* and then in the morning when I had to get up*,* I would finally fall asleep. My bedtimes were all over the place*,* sometimes it would go well for a day*,* and then for about a week and a half it was very inconsistent*,* which just kept going on like that. [Interviewer: So*,* by doing sleep restriction*,* you were able to bring some more regularity into your sleeping pattern? ] Yes*,* I now managed to stick to it*,* that was very valuable.”* – Patient 5 (male, 39 years). Despite its perceived effectiveness, patients also reported challenges with sleep restriction: it was very difficult to maintain, especially on weekends or days off due to a lack of obligations in the morning. Some patients also struggled with the extended waking hours, as they were no longer able to go to bed early as a way to escape daytime difficulties but instead had to “sit out the day”. One patient mentioned negative effects during the initial phase, such as severe fatigue and feeling overstimulated. For one patient, shift work hindered the implementation of fixed bedtimes.

##### Relaxation

Treatment components that essentially focused on relaxation were most consistently valued. All patients mentioned that an evening ritual to wind down the day had been very beneficial, which was similarly valued in quantitative ratings (M = 7.11 ± 1.94). An evening ritual helped reduce arousal and stop dwelling on negative thoughts: *“I think that winding down the day and moments of rest during the day helped. That was very important. For example*,* to not watch television or the news*,* otherwise my mind would end up in chaos. … This way*,* I kind of protect myself from all the troubles and turmoil in the world.”* – Patient 2 (male, 52 years). Lifestyle adjustments to integrate more moments of rest and relaxation during the day also helped reduce worrying and improve sleep. The specific ways to increase relaxation varied. Some patients found that explicit relaxation exercises such as provided in session 3 or other (guided) meditative practices worked well, although patients often combined this with listening to music “to avoid racing thoughts”. However, the majority found these introspective exercises not helpful, sometimes even leading to more tension, which was often linked to mental restlessness: *“I have severe ADHD and had no interest at all in that stuff about mindfulness. No*,* I really didn’t feel like doing that. Sounds a bit harsh*,* but that’s really what it comes down to.”* – Patient 1 (male, 28 years).*“Well I tried it*,* and I was like ‘ok*,* I’m lying in bed*,* I feel my toes’ etc.*,* but that is too vague for me. It didn’t really help me relax and instead I was lying there worrying about why I felt my shoulder twitching. … Bodily relaxation exercises are very difficult for me*,* but actually actively doing something to relax; that was really valuable. But if I must pay attention to my body*,* in my mind*,* that vague stuff*,* I can’t get there.” –* Patient 3 (female, 34 years). Instead, these patients benefited from more active forms of relaxation, such as listening to a podcast, creative activities, or physical exercise: *“Mainly taking more time for myself*,* moments of rest*,* of relaxation. Like scrapbooking*,* that’s something that keeps me busy*,* relaxes me*,* where I don’t have to think about other things for a while. So. relaxing activities*,* walking as well*,* etc. These are valuable things to keep yourself occupied during the day*,* so you don’t dwell on things*,* get some sense of fulfillment*,* and stretch time a bit to go to bed later.”* – Patient 3 (female, 34 years).

##### Rumination and (sleep-related) cognitions

The structured ‘worry time’ exercise and other thought-blocking techniques were consistently perceived as unhelpful during the interviews, as also reflected in the relatively low effectiveness ratings in the questionnaire (M = 5.69 ± 2.47). *“Worry time? Let’s just say those 15 minutes were not enough for me. I would think ‘ok*,* I’m going to sit down and worry now’ but then my mind started racing very fast. … So in that moment I was thinking this and this and this is bothering me*,* and blah*,* blah*,* blah*,* but. that didn’t help*,* let’s just say that.” –* Patient 3 (female, 34 years). The majority also found the exercises to restructure negative sleep-related cognitions to be unhelpful, mostly because they experienced an abundance of negative thoughts, also unrelated to sleep, that were very hard to challenge and rephrase: *“No*,* not for me*,* because I struggle a lot with my thoughts and seeing things positively. … My thoughts are all over the place*,* so that session [session 4] did not help me. … I really struggled with this*,* because my thoughts are mostly negative*,* they are always negative actually*,* which makes it really hard to come up with something positive.” –* Patient 3 (female, 34 years). However, some patients did consider this technique helpful, as also reflected by quantitative ratings (M = 6.41 ± 2.06), although difficult and requiring practice and time: *“I did find that one [session 4] helpful*,* yes definitely. It was difficult*,* but I noticed in hindsight that the more you try*,* the more I noticed a difference*,* now I don’t focus on it as much: ‘I can sleep*,* I’ll try*,* and we’ll see’.”* – Patient 4 (female, 22 years).

##### Other treatment components

Regarding other treatment components, all except for one interviewee reported that the suggested strategies for adjusting their bedroom surroundings were not helpful, in line with relatively low quantitative ratings (M = 5.93 ± 2.43). They either already had applied this, or their bedroom did not allow for such changes. Furthermore, several patients mentioned that the final session, which involved a summary of all prior sessions and planning for the future, helped to recall and reinforce what they had learned, as they had forgotten some of the information from earlier sessions. However, its perceived benefit only became apparent upon reflection after explicit inquiry during the interviews.

**Table 4:** Quantitative treatment evaluation data of overall satisfaction with, effectiveness of, and maintained adherence to iCBT-I (components)

iCBT-I components	Treatment evaluation T1: Effectiveness				Treatment evaluation T2: Adherence				
	Interview subsample (*n* = 5)		Total sample (*n* = 19)		Interview subsample (*n* = 5)			Total sample (*n* = 21)	
	M (*SD*)	Range	M (*SD*)	Range	M (*SD*)	Range		M (*SD*)	Range
Session 1									
Lifestyle adjustments	7.00 (2.55)	3–10	6.53 (*2.43*)	3–10	4.60 (2.51)		1–7	5.81 (*2.04*)	1–10
Bedroom surroundings	5.75 (3.40)	1–9	5.93 (*2.43*)	1–10	5.80 (3.35)		2–10	5.57 (*2.58*)	1–10
Evening ritual	7.20 (2.17)	4–10	7.11 (*1.94*)	4–10	6.20 (3.35)		1–10	5.57 (*2.89*)	1–10
Session 2									
Fixed bedtimes	7.40 (1.82)	5–10	7.00 (*1.79*)	4–10	4.60 (2.51)		1–8	5.25 (*2.22*)	1–10
Sleep restriction	7.20 (1.79)	5–10	6.06 (*2.59*)	1–10	5.80 (2.95)		2–10	5.25 (*2.99*)	1–10
Session 3									
Relaxation	7.25 (2.22)	5–10	6.33 (*1.99*)	3–10	4.40 (3.97)		1–10	3.42 (*2.71*)	1–10
Rumination	5.80 (3.44)	1–10	5.69 (*2.47*)	1–10	2.60 (1.82)		1–5	2.89 (*2.42*)	1–10
Session 4									
Sleep-related cognitions	5.60 (2.88)	2–9	6.41 (*2.06*)	2–10	5.60 (3.21)		1–10	4.16 (*3.24*)	1–10
Session 5									
Future plan	7.25 (2.87)	3–9	6.17 (*2.08*)	3–9	4.50 (4.36)		1–10	4.07 (*2.84*)	1–10
Overall satisfaction iCBT-I	6.40 (1.52)	5–8	5.79 (*1.40*)	3–8					
Satisfaction online guidance	7.80 (1.30)	6–9	7.21 (*1.47*)	4–9					

Note. Quantitative insights were assessed using a 10-point Likert scale aimed at perceived effectiveness at T1 (1 = not at all effective, 10 = highly effective) and long-term adherence after completing the intervention at T2 (1 = never performed again, and 10 = performed daily). Additional summary measures (medians and interquartile ranges) are provided in Supplementary Table S1

#### Treatment delivery

##### Online treatment

All interviewees considered both the web-based platform and sleep diary-app easy to use. Additionally, all patients experienced the online treatment delivery positively, and all but one patient (who preferred hybrid treatment) preferred to receive treatment like this over in-person delivery. They valued the flexibility to combine treatment with other obligations such as work, and reduced logistical burdens of time, costs, and hassle of travel. *“I thought it [online treatment] was really great*,* yes*,* very nice. At first I thought I had to follow a lesson each week on [a fixed schedule]. But I liked it way better with the online modules*,* because with your private life. you could rearrange things*,* do your own thing*,* that makes it easier. [Interviewer: did that make it easier to continue as well? ] Yes*,* definitely. We [likely referring to her generation] are handy with all the apps and phones*,* I really liked this a lot better than having to write on paper or constantly go back and forth. I would have dropped out real quick if that had been the case.”* – Patient 4 (female, 22 years). Some found information easier to process and remember this way, being able to go through it at their own pace, and felt it contributed to a feeling of ownership and engagement: *“I liked that it wasn’t just talking and then being sent off to apply it*,* but that you got the chance to really do it yourself: to read*,* to fill out*,* to keep track. That gives a sense of responsibility*,* instead of someone else keeping track of everything.” –* Patient 3 (female, 34 years). In addition to these practical benefits, some patients preferred the low-key nature of online treatment over more formal, in-person appointments, and one patient also emphasized that the online format, against expectation, facilitated more frequent and close contact with the therapist. Although all patients appreciated the online treatment, one patient mentioned feeling uncomfortable having to see herself on screen during videocall appointments. Furthermore, some patients offered concrete suggestions for improving the iCBT-I intervention i-Sleep, including the ability to revisit their own notes and answers after completing a session, and the use of a single platform for both the iCBT-I sessions and sleep diary. No other suggestions for improvement were proposed.

##### Therapist guidance

Patients emphasized that therapist guidance was imperative in the online treatment. In line with positive quantitative ratings of satisfaction with the online guidance (M = 7.21 ± 1.47), all interviewed patients experienced contact with the iCBT-I therapist as positive, appreciating the approachability of the therapist, the attention given to their personal situation, and the collaborative approach to making individual adjustments: *“I could easily contact her*,* send a message via the Sleep Registry if something wasn’t clear or when things weren’t going well for me. I thought she was a very nice therapist. She also tried to find solutions with the sleep restriction*,* with my rotating shifts. I really appreciated that. [Interviewer: Was there anything you missed in the contact with the therapist? ] No*,* it was actually very responsive communication*,* I thought it was perfect*,* really great.”* – Patient 4 (female, 22 years). The amount and frequency of guidance provided by the therapist was also deemed sufficient by all. The current approach provided enough support, and some specifically mentioned that guidance also should not be too often: *“Yes it was enough. It also shouldn’t be too much*,* then it becomes a bit of a burden. I thought it was good just as it was*,* not too much*,* but sufficient. I think I would have disliked it if I would have had to call every week. But this way*,* when you completed a session*,* you received the written feedback every week*,* that was good*,* I think that’s better than a videocall-appointment each week.”* – Patient 5 (male, 39 years).

###### Value of written and videocall guidance

The two types of guidance (i.e., written feedback and videocall appointments) were evaluated differently regarding their value and necessity. For the majority, written feedback was not considered strictly necessary for successfully completing iCBT-I, but they did not perceive receiving the feedback negatively or as a burden. Some found the feedback helpful in providing a sense of support and motivation, knowing that someone was actively monitoring their progress, and by getting personalized advice to better apply the techniques to their individual situation. The videocall appointments were considered imperative for following iCBT-I by all patients.*“[Interviewer: Do you think you could have completed i-Sleep without the videocall guidance? ] I don’t think so. You can click through it all nicely*,* but you need to have some sort of contact with the therapist. … That really must be part of it. I think it should be both [written feedback and videocalls]. Some things can be done through writing*,* but sometimes things don’t come across well in writing*,* and then the videocalls are a better way to discuss this. I think the most important thing is the videocall guidance*,* you can simply say more than by just writing*,* and sometimes I also just don’t feel like writing it all down in a long piece of text.”* – Patient 4 (female, 22 years). These appointments helped them stay motivated and continue with the intervention, again because of a sense of support knowing that a “real person” was actively involved, and by having a set appointment as incentive: *“You need to have personal contact*,* whether by phone or videocall*,* but having real contact where you actually have an appointment. [Interviewer: So*,* you preferred also having the videocalls*,* instead of solely receiving written feedback? ] With only the written feedback? No*,* then it would have been very difficult*,* because I often ignore my emails. I think I didn’t even notice most of those messages coming in.”* – Patient 5 (male, 39 years). The videocall appointments were additionally perceived as an important moment to reflect on the content of and progress with the iCBT-I components and personally discuss this with the therapist to get more individually tailored feedback. *“Having completed a couple of sessions in a row*,* for instance*,* I had completed one*,* two and three*,* so then we could briefly summarize those three*,* as there was lots to fill out in those sessions. And it also adds a more personal touch*,* like. ‘How is it all going?’*,* just having a personal chat. But maybe I’m a bit old-fashioned that way*,* feeling like you should still have some personal contact. [Interviewer: Do you think you could have completed i-Sleep without those videocall appointments? ] No*,* that did make it easier to continue*,* by having that personal element.”* – Patient 2 (male, 52 years).

###### Necessity of reminders

A spontaneously mentioned theme in the interviews was the necessity of reminders from the therapist as a crucial and helpful component for adhering to and continuing iCBT-I. Log data underscored the high need for reminders, showing that almost all patients (97%) required at least one reminder during the intervention, and an average of 5.13 reminders (± 2.50) were needed per patient. The need for reminders was often attributed to forgetfulness due to chaos in daily life, and being reminded furthermore created a sense of accountability and necessary pressure to complete the sessions: *“I liked the fact that you’re responsible for it yourself*,* but mainly*,* I liked that she would follow up with me. Because it quickly tends to fall off track for us*,* but if you were then called with a reminder*,* it would put some more pressure on me to actually do it.”* – Patient 3 (female, 34 years).

#### Integration of iCBT-I and BPD treatment

##### Timing of iCBT-I relative to BPD treatment

BPD treatment paths varied between individuals, regarding type of BPD treatment and timing of iCBT-I with respect to BPD treatment. Since patients’ perspectives were limited to their own unique treatment trajectory, some found it difficult to comment on hypothetical alternatives and provide a definitive opinion on the optimal timing of iCBT-I relative to their BPD treatment. Patients that finished iCBT-I during the waiting period for BPD treatment thought this timing to be beneficial, as the prospect of simultaneous treatment seemed too much of a burden, and it allowed them to engage with some “lighter” issues first, during otherwise inactive waiting-time. For those with partly overlapping sleep and BPD treatment, the combined workload was experienced as intensive and tiring by some, although one patient also appreciated being able to work on both sleep and BPD symptoms simultaneously.

##### Necessity of targeted yet integrated sleep treatment

All patients mentioned that specific attention for treatment of sleep problems was helpful. They noted that while sleep and BPD symptoms are connected, their sleep problems are independent and require targeted treatment. Some emphasized they therefore appreciated receiving iCBT-I separately from their BPD treatment for a more explicit focus on sleep. However, most patients mentioned that explicit attention to sleep should be integrated in BPD care in general. *“Now at [mental healthcare center]*,* it’s really focused on borderline*,* but on top of that*,* I also have sleep problems*,* and multiple other co-occurring difficulties going on*,* but that’s all on hold. It’s not being combined now*,* so essentially*,* you’re in therapy for your borderline for a few years*,* and then when that’s over*,* you still have to deal with your sleep*,* and then with this*,* and then with that. If it could all be a bit more integrated*,* I think you’d kill two birds with one stone*,* that would be better.” –* Patient 3 (female, 34 years). Some also mentioned logistical benefits of integration, such as avoiding the need to navigate between multiple platforms and different therapists, and benefits extending beyond initial treatment, as some patients thought that integration of sleep treatment into their subsequent BPD treatment would have helped to better maintain the achieved improvements after following iCBT-I. However, patients also acknowledged that simultaneous treatment could become overwhelming due to the complexity of BPD and other comorbidities.

### Maintenance

#### Long-term adherence to iCBT-I components

All patients mentioned that they continued to incorporate the varying relaxation exercises they learned during iCBT-I. Winding down with an evening routine and implementing activities to relax, such as exercise and creative activities, were most consistently maintained. The majority of patients valued the techniques related to sleep restriction the most, especially avoiding going to bed too early and maintaining a consistent sleep-wake rhythm, and continued to apply them after the program. *“For me mainly the sleep restriction*,* that was really valuable and I still apply this … When I’m very tired but it’s only 8:00 pm*,* I say to myself ‘Yeah*,* it’s a bit too early to go to sleep now isn’t it.’. So*,* for me*,* the sleep restriction was really helpful; that’s something I want to continue in the future*,* that can really help; staying awake longer.”* – Patient 3 (female, 34 years). This sustained use of techniques was also reflected in the quantitative data, with components such as lifestyle adjustments (M = 5.81 ± 2.04), evening ritual (M = 5.57 ± 2.89), fixed bedtimes (M = 5.25 ± 2.22), and sleep restriction (M = 5.25 ± 2.99) receiving the highest long-term adherence ratings at 8-month follow-up (Table 4). Some patients also mentioned they no longer (actively) applied some techniques, as their insomnia symptoms had improved.

#### Future intentions and challenges with long-term adherence

Patients also reflected on the future and indicated that they wanted to continue using the implemented iCBT-I techniques. They also planned to review i-Sleep in the future, if necessary, to refresh or re-attempt applying techniques they initially skipped, since extensive behavioral change requirements led most patients to strategically focus on some key aspects to keep iCBT-I manageable and feasible. While most patients still actively applied some techniques, they also acknowledged challenges and struggles with maintaining change in the long-term for instance regarding adherence to avoiding phones in bed or waking up at fixed times on weekends. It was also noted that their focus on applying the techniques faded as therapist guidance seized after iCBT-I and was not integrated into subsequent BPD treatment. Challenges with sustained behavioral change were also reflected by quantitative evaluations at follow-up, indicating generally poor to modest maintained adherence (average rating of components ranging from 2.89 to 5.81 on a scale of 1 to 10, Table 4). Despite these challenges, all interviewed patients stated they would recommend the iCBT-I intervention i-Sleep to others with similar complaints, emphasizing it was very helpful to focus specifically on sleep and that the intervention contains plenty of strategies to address individual needs.

## Discussion

In this mixed-method process evaluation, we aimed to identify key determinants of successful implementation, and underlying processes and mechanisms of guided iCBT-I for patients with BPD. Our findings demonstrated the potential of an online treatment format and the importance of personalized and responsive therapist guidance. We identified particularly effective as well as more challenging treatment components and revealed a strong wish among patients for targeted treatment of sleep disturbances, preferably well-integrated within broader BPD treatment trajectories.

### Attrition and therapist-guided treatment delivery

Online interventions for patients with BPD are relatively new. A recent meta-analytic review, while demonstrating potential for online interventions in this patient group, also stated concerns regarding the high attrition rates [30]. In our study, the rate of attrition was 30%. This is substantially lower than the attrition rates of 53–63% in studies on fully automated iCBT-I interventions in people with (symptoms of) depression [31, 32]. A recent process evaluation of i-Sleep in individuals with (sub-)clinical anxiety- or trauma-related mental health complaints, reported comparable attrition rates of 31% in patients from specialized mental healthcare, but attrition was notably lower in sub-groups with less severe complaints (16%-19%) [20]. Attrition rates in iCBT-I have also been shown to reduce with online therapist guidance (vs. no support), especially for people with higher severity of psychopathology [33]. In our study, all patients had positive experiences with the online approach and the majority even expressed a clear preference for it over traditional in-person treatment, citing advantages due to its flexibility, increased engagement and ownership, and low-key nature. Therapist guidance was explicitly mentioned as a critical component of the online intervention. The videocall appointments, which we added to the original i-Sleep protocol to address anticipated difficulty with adherence and continuation, were deemed imperative by all patients to enhance motivation and provide personal contact. In contrast, in previous iCBT-I studies in psychiatric populations with no additional videocall guidance, part of the patients and therapists expressed the wish for more personalized support and personal contact by (video)call [20, 34]. Our findings on the value of therapist support align with the Supportive Accountability model, which posits that human support enhances adherence to digital interventions through a sense of accountability to a supportive and knowledgeable coach [35]. Our study moreover revealed the crucial importance of reminders from the therapist, which was spontaneously mentioned by all patients and provided a crucial sense of accountability to continue with and adhere to the intervention, especially given their often chaotic daily lives. The need for reminders during iCBT-I was notably higher in our BPD patient sample (5.13 reminders needed on average) compared to individuals with (sub-)clinical anxiety- or trauma-related complaints (2.5 reminders needed on average) [20]. Importantly, the addition of videocall appointments did not necessarily lead to higher therapist time investment (137 min in our study vs. 156 min in the comparable process evaluation by Reesen and colleagues [20] in which i-Sleep was provided without videocall guidance). The more direct, verbal communication may have saved time that would otherwise have been spent on written feedback, which requires careful formulation to avoid misinterpretation and address individual patient needs. Together, this indicates that an online approach to CBT-I is very well feasible in BPD patients, although a guided approach, with proactive reminders and extra videocall guidance next to written feedback, is crucial for patient retention and may represent a more efficient use of therapist time.

### Effectiveness of iCBT-I in patients with BPD

The process evaluation findings regarding effectiveness were strongly aligned with the clinical outcomes of the overall RCT [16]. As in the RCT, patients also qualitatively did not perceive a clear effect of iCBT-I on BPD symptom severity, and found it difficult to isolate the intervention’s impact from their BPD treatment. Moreover, given the pervasive and enduring nature of BPD symptomatology, patients expected alleviation of overall symptom severity to take more time. However, patients did report that improvements in sleep led to a reduction in specific symptoms like irritability and restlessness, which is in line with the RCT showing significantly less morning tension and fewer anger attacks in patients in the iCBT-I condition. This suggests that iCBT-I selectively improved some BPD symptoms closely linked to arousal. Regarding sleep, all patients experienced clear positive effects on sleep, such as reduced insomnia symptoms. Likewise, the RCT demonstrated significant moderate to large improvements in insomnia severity, sleep quality, and other sleep continuity measures. Together, these findings indicate a strong concordance between patients’ in-depth subjective experiences and quantitative outcomes of the main trial, strengthening the validity of our results and indicating that iCBT-I effectively treats insomnia and specific arousal-related symptoms in patients with BPD.

### Considerations for optimal implementation of iCBT-I for patients with BPD

Our findings have several implications for the implementation of iCBT-I for patients with BPD. First, the most effective component was perceived to be sleep restriction, and particularly the part of sticking to fixed bedtimes. While patients found this a challenging component to implement, it was also considered the most effective for improving their sleep and establishing a much-needed routine. Notably, most patients did not strictly adhere to the time prescribed by sleep restriction guidelines but often focused more on the regularity of their bedtimes. Especially for patients with highly variable bed and rise times and those that spent very long times in bed prior to iCBT-I, even more lenient sleep restriction approaches required major behavioral change. Previous studies also suggest that rigid sleep restriction may not be essential for achieving significant improvements. In patients with bipolar disorder bedtime regularization was shown to be sufficient for improving sleep [36]. Additionally, recent research on sleep compression, which prioritizes fixed bedtimes and then gradually shortening the time in bed, also led to large improvements in insomnia severity while being more feasible and tolerable than sleep restriction [37, 38]. Although one patient in our study reported a short-term exacerbation of fatigue and anxiety during early sleep restriction, these findings indicate that these more subtle approaches may be sufficient and more feasible while further reducing the chance of short-term side-effects.

This process evaluation moreover highlights the importance of integrating relaxation strategies, while also demonstrating the need for a personalized approach. While more traditional, introspective relaxation exercises were helpful for some, these were ineffective or even caused increased tension and rumination for others. Instead, patients benefited more from incorporating lifestyle changes such as active relaxing activities throughout the day. Although there was no one-size-fits-all approach, establishing a dedicated evening winding-down routine seems to be a crucial treatment component. This routine provided a necessary buffer between daily stressors and the process of falling asleep, thereby managing pre-sleep hyperarousal. This may be particularly important with BPD, which is associated with high states of arousal and the overwhelming experience of emotional cascades and flooding rumination. This may also explain why more passive and introspective relaxation exercises can be counterproductive by exacerbating rumination. Similarly, cognitive components of iCBT-I, like thought-blocking and reappraisal techniques, may be less effective due to being overwhelmed by intense negative BPD- (and trauma-) related thoughts and emotions in the process. For patients struggling with clear insomnia-related rumination, more intensive guidance seems necessary to demarcate BPD- and insomnia-related cognitions.

Finally, our process evaluation demonstrated the clear desire for specific attention to sleep disturbance in the broader BPD care pathway. Preferences regarding the timing of sleep treatment within the BPD treatment trajectory differed, with some patients experiencing an overlap in treatments as overwhelming and too burdensome. Nevertheless, a coordinated or integrated approach was favored by most patients, which resonates with preferences expressed in a comparable recent i-Sleep process evaluation by the subgroup of patients receiving specialized mental healthcare [20]. Integration of sleep treatment into BPD treatment was also expected to have helped with maintenance of the achieved improvements after following iCBT-I. From both a practical and theoretical perspective iCBT-I is preferentially delivered during waiting list periods for BPD treatment. This could optimize treatment gain in an otherwise inactive period and maximize sleep-dependent consolidation of adjusted maladaptive memories and newly acquired skills during subsequent BPD treatment. Yet, the general difficulty with sustained behavioral change, as reflected by the quantitative maintenance results, emphasizes the need for continued attention to (relapse of) sleep disturbances. Ideally this could be done by the same treatment team and aligned with sleep-focused treatment modules of BPD treatments as they exist in e.g. dialectical behavior therapy (DBT) and systems training for emotional predictability and problem solving (STEPSS).

### Limitations and strengths

Our process evaluation has several limitations and strengths. The context of the study as part of the RCT differs from a naturalistic clinical setting. This likely impacted patient perspectives on the integration of iCBT-I and subsequent BPD treatment and their reasons for participation and continuation. Additionally, because participants voluntarily participated in a trial involving an online intervention, our findings on the desirability of online CBT-I may not generalize to the broader BPD population. Patients with a strong preference for face-to-face treatment may have chosen not to participate in the trial. Moreover, our study focused mainly on patient perspectives. Future studies in clinicians and other stakeholders could shed light on other potential barriers and facilitators to implementation of iCBT-I for patients with BPD in clinical practice. Additionally, iCBT-I guidance was provided by a single therapist, who was also the main researcher (SvT), limiting generalizability of the results and introducing a potential for bias. Importantly however, qualitative interviews were conducted by an independent researcher and interpretation of qualitative data was carried out collaboratively involving multiple researchers. Reversely, this single-therapist approach also provided a unique benefit by offering a complete and cohesive overview of the intervention process among all patients. Furthermore, the generalizability of our findings is limited by the small subsample of qualitative interviews, which also only contained intervention completers and was relatively homogeneous regarding socioeconomic background. Consequently, qualitative insights predominantly reflect the perspectives of patients who maintained engagement, potentially omitting specific barriers or needs regarding feasibility and guidance experienced by those who discontinued treatment or with different socioeconomic backgrounds. Moreover, although part of the patients that discontinued iCBT-I did continue participation, 6 patients (20%) dropped out of the RCT entirely and therefore did not complete the treatment evaluation questionnaires. However, the study’s mixed-method approach, with qualitative exploration of patient perspectives complemented with quantitative treatment evaluation and therapist guidance insights, provide important broader context. Our results furthermore were in strong concordance with the clinical trial results [16], supporting the validity of our conclusions even with a limited number of interviews. Finally, our study is the first to evaluate iCBT-I in patients with BPD, a complex and often overlooked patient population for whom there is a clear need for effective and accessible new treatment strategies, and provides important considerations for its implementation (see Box 1).

**Box 1:** Considerations for implementation

• **An online approach with personalized guidance**: The online approach of iCBT-I is a promising and desired modality, although its success may be contingent on personalized, responsive guidance that includes proactive reminders, and additional videocall guidance.	
• **Emphasize behavioral strategies**: Clinicians should not be hesitant to employ behavioral strategies such as ***sleep restriction***. While challenging, restricting time in bed to ultimately establish a fixed bedtime schedule may be most effective. For patients with severely dysregulated sleeping patterns, a more lenient or gradual restriction of time in bed may be a more feasible but effective alternative. Another key behavioral strategy is the use of ***tailored relaxation techniques***. Particular attention to establishing and maintaining an evening winding-down routine seems crucial, with specific relaxation activities tailored to individual patient preferences and their often limited capacity to introspectively manage emotional distress and rumination.	
• **Well-guided use of cognitive techniques**: Despite the tendency to be generally overwhelmed by BPD-related thoughts and emotions, cognitive strategies can still be beneficial for patients struggling with insomnia-related rumination, provided that the focus lies clearly on the sleep-related cognitions.	
• **Integrate sleep treatment into BPD care**: to address the expressed need for dedicated treatment of sleep problems, a well-integrated approach is required, from initial treatment through long-term maintenance. Further evaluation of implementation processes from the perspective of clinicians and organizations can provide further directions in this crucial, yet often overlooked, aspect of care while minimizing treatment burden.	

### Conclusion

Patients with BPD experienced guided iCBT-I as a beneficial and much-needed intervention, which, due to its relatively short duration and little therapist investment, offers a promising approach to alleviate insomnia and specific arousal-related symptoms. When provided with personalized (videocall) therapist support, focused on key behavioral strategies tailored to the unique needs of this patient population, and well-integrated into broader BPD care, this accessible and scalable intervention holds great potential as a new treatment strategy for BPD and should be considered an integral part of routine BPD care.

## Supplementary Information

Below is the link to the electronic supplementary material.

Supplementary Material 1

## Data Availability

The data used in this study are not publicly available. Deidentified quantitative data may be available upon reasonable request (including a study proposal). Requests can be sent to the principal investigator (HvM: h.j.vanmarle@amsterdamumc.nl), are subject to review by the research team and management of the department, and require a formal data access agreement.

## Abbreviations

BPD: Borderline personality disorder
BPDSI: Borderline Personality Disorder Severity Index-5 4-week version
CBT-I: Cognitive behavioral therapy for insomnia
DBT: Dialectical behavior therapy
iCBT-I: Online cognitive behavioral therapy for insomnia
ISI: Insomnia Severity Index
MINI: Mini-International Neuropsychiatric Interview
PD: Personality disorder
PTSD: Post-traumatic stress disorder
RCT: Randomized controlled trial
RE-AIM: Reach, Effectiveness, Adoption, Implementation and adherence, Maintenance
SCID-5-P: Structured Clinical Interview for DSM-5 Personality Disorders
STEPPS: Systems training for emotional predictability and problem solving
