# Supplementary material for: A mixed-method process evaluation of guided online cognitive behavioral therapy for insomnia in patients with borderline personality disorder
Source: BMC Psychiatry. 2026 Feb 12;26:244. doi: 10.1186/s12888-026-07859-8 (PMC12997894; doi:10.1186/s12888-026-07859-8)
Supplement: Supplementary file 1 — Supplementary Material 1 [file 12888_2026_7859_MOESM1_ESM.pdf]

Supplementary material

Title: A mixed-method process evaluation of guided online cognitive behavioral therapy for insomnia in patients with borderline personality disorder

Shanna van Trigt[a,b,c], Femke van Nassau[d], Tanja van der Zweerde[c,e], Eus J. W. van Someren[a,g,h,i], Annemieke van Straten[b,c], Hein J. F. van Marle[a,f,g,j]

[a] Amsterdam UMC, location Vrije Universiteit, Psychiatry, Amsterdam, the Netherlands

[b] Amsterdam Public Health, Mental Health program, Amsterdam, the Netherlands

[c] Vrije Universiteit Amsterdam, Clinical, Neuro and Developmental Psychology, Amsterdam, the Netherlands

[d] Department of Public and Occupational Health, Amsterdam Public Health research institute, Amsterdam UMC, location Vrije Universiteit, Amsterdam, the Netherlands

[e] Wagner Psychologists, Utrecht, the Netherlands

[f] GGZ inGeest Mental Health Care, Amsterdam, The Netherlands

[g] Amsterdam Neuroscience, Mood, Anxiety, Psychosis, Sleep & Stress program, Amsterdam, the Netherlands

[h] Department of Sleep and Cognition, Netherlands Institute for Neuroscience, an Institute of the Royal Netherlands Academy of Arts and Sciences, Amsterdam, the Netherlands

[i] Department of Integrative Neurophysiology, Center for Neurogenomics and Cognitive Research, Amsterdam Neuroscience, Vrije Universiteit, Amsterdam, the Netherlands

[j] ARQ National Psychotrauma Center, Diemen, the Netherlands

Acknowledgements

Funding by the Netherlands Brain Foundation (grant number: DR-2019-00319).

Corresponding author: Shanna van Trigt, s.vantrigt@amsterdamumc.nl, Amsterdam UMC, location Vrije Universiteit, Psychiatry, De Boelelaan 1117, Amsterdam, the Netherlands

Table of contents

1. Topic-guide qualitative interview 3

2. Treatment evaluation questionnaires 7

   2.1. At T1 (2-month follow-up): perceived effectiveness 7

   2.2. At T2 (8-month follow-up): long-term adherence 8

3. Baseline assessments 9

4. Codebook 10

5. Supplementary results 16

References 17

1. Topic guide qualitative interview

<u>Opening</u>
Introduction:
Good morning/afternoon, I am [...]. from the 'Better nights, better days?' study. Thank you for making time for this interview about your experiences with i-Sleep.
(If necessary: suggest finding a quiet place for the conversation).

Goal of the conversation:
First of all, thank you very much for agreeing to participate in this interview. During your participation in the 'Better nights, better days?' study, you completed the digital sleep module i-Sleep. The goal of this conversation is to discuss your experiences with i-Sleep, to review to what extent i-Sleep has or has not helped you, and how the module can best be offered in a healthcare setting. There are no right or wrong answers, honesty helps us the most, so please feel free to share openly.

Consent for recording:
With your permission, the conversation will be recorded, after which it will be anonymized. The conversation will be literally transcribed, and the recording will be deleted. Everything you tell us in this interview cannot be traced back to you. We only record the audio, not the video of the call. Do you have any questions? Do you give your consent to record this conversation?

If Yes: start recording

Just to confirm while recording: do you, [subject identification number], give your consent to record this conversation?

<u>Main interview questions</u>
General questions about the study:
We will start with some general questions.

- Why did you decide to participate in the study? What did you hope to achieve by participating?
- What were your expectations regarding i-Sleep?
- About the content of the module?
- About the outcomes of the module?

Using the i-Sleep module:
Now we will continue with questions about using i-Sleep.

- Can you tell me about your experiences with the i-Sleep module? (e.g., did you find it easy to use? Did you find the information useful?)
- Why did you finish it / not finish it?
- *If not finished*: Do you have any ideas about what could have helped you to want to / be

able to finish i-Sleep?

The i-Sleep module consisted of five sessions, and in each session, new topics were discussed. I will name these topics. Can you indicate to what extent you used a component, and whether you found it valuable?
(Name each session's components and ask to what extent they were used and valuable. If necessary, ask for further explanation and/or specific examples.)

1. The first session covered sleep hygiene, which means adjusting the bedroom surroundings, winding down the day, incorporating moments of rest during the day, and making lifestyle adjustments (e.g., exercising more, reducing coffee and alcohol).
2. In the second session, sleep restriction was introduced, where you reduced your time in bed to increase sleep pressure.
3. In the third session, you learned techniques to cope with worrying thoughts, such as the 15-minute worry-time, thought-blocking (repeating the word 'the'), and relaxation exercises.
4. The fourth session was about reframing negative, unhelpful thoughts about sleep.
5. The last session was a kind of review of which components of the module were effective for you and how you wanted to continue using them to sleep better.

- Which components were most helpful to you? And why?
- Which components were not or least valuable to you? And why?
- Did you miss anything in the i-Sleep module that you would have liked to see in the program?

Digital method:
Now I would like to talk to you about the fact that this was an online module.
- What did you think of the online method used (the online sessions in combination with filling out the sleep diary, the digital feedback, and the video call appointments) during the i-Sleep module? *(follow-up questions important)*
- Did you find the website with the online i-Sleep sessions easy to use? Can you explain?
- What went well / less well? (e.g., logging in, completing a session, requesting feedback, filling out the sleep diary/viewing your data, etc.)
- How would you have preferred to follow i-Sleep, digitally, as in this study, or face-to-face, or a combination of both? Can you explain why?

Therapeutic relationship and feedback:
The following questions are about your therapist and the online guidance of i-Sleep, which includes the written feedback messages via the website and the video call appointments you had.
- What did you think of the contact with your therapist? Why? *(follow-up questions important, e.g., about sense of personal contact, accessibility, help with*

motivation/adherence, consideration of personal circumstances)

- Did you miss anything in your contact with your therapist? If so, what was that?
- Did you feel that the written feedback helped you further to reduce your complaints? Why or why not?
- Did you feel that the video call appointments helped you further to reduce your complaints? Why or why not?
- Do you think you could have completed i-Sleep without the written feedback? Why or why not?
- And without the video call appointments? Why or why not?
- What did you think of the frequency of the written feedback? And of the video call appointments?
- What did you think of the content of the written feedback? and of the video call appointments?
- Would you have preferred to receive guidance in a different way?
- For participants who did not finish i-Sleep: Could a different method of guidance have made it possible for you to finish i-Sleep?
- Do you have any suggestions to improve the guidance during i-Sleep?

Perceived effectiveness:

Now I would like to talk to you about the effect that following i-Sleep has had for you.

- Did i-Sleep help you to sleep better?
- What has mainly improved about your sleep? (e.g., falling asleep faster, waking up less often at night, sleeping longer, feeling more rested during the day, etc.)
- Did i-Sleep help you in reducing your borderline personality symptoms?
- What has mainly improved? (e.g., fewer mood swings, impulsivity, or feeling overwhelmed by emotions or worrying in the evening)
- What has influenced this the most? (e.g., less time lying awake in bed, less fatigue during the day, a more fixed rhythm/more regularity)

Combination of iCBT-I and BPD treatment:

The following questions are about following i-Sleep in combination with your other mental health care treatment(s) for borderline personality symptoms.

- Did you follow i-Sleep during the waiting period for BPD treatment, at the same time as BPD treatment, and/or after BPD treatment?
- What did you think of this timing?
- What do you think is the best time to follow i-Sleep? Can you explain why?
- Did i-Sleep help you in following your other mental health care treatment? How did you notice that?
- Did you prefer receiving a separate treatment for sleep problems, or would you have preferred to do this together, integrated into your other BPD treatment? Can you explain why?
- *For participants who did not finish i-Sleep*: Did the combination or timing of i-Sleep with

your other mental health care treatment influence your stopping/not finishing i-Sleep?
- Did you notice any other things in your daily life that were the result of following i-Sleep? (e.g., regarding your mood, lifestyle)

Maintenance:
Now I would like to talk to you about whether you still use i-Sleep or would like to continue using it in the future.

- Do you still use the i-Sleep information or techniques that you learned for your sleep? If so, which ones and why? If not, why not?
- Would you like to use i-Sleep again in the future?
- Would you recommend the i-Sleep module to others?

Closing
Those were all my questions for you.

- Is there anything else you would like to share about i-Sleep that we haven't discussed yet?
- Do you have any further comments/questions?

As I mentioned at the beginning of the conversation, we will use your experiences with the ultimate goal of offering i-Sleep in the most optimal way in clinical practice. We will pseudonymize all your feedback. Thank you very much for your participation in 'Better nights, better days?' and for sharing your experiences during this conversation. Your insights are incredibly valuable to our research. I wish you a very nice day.

2. Treatment evaluation questionnaires

2.1. At T1 (2-month follow-up): perceived effectiveness

Item	Response option
**How satisfied were you with the online i-Sleep module?**<br>Rate your satisfaction on a scale of 1 (very dissatisfied) to 10 (very satisfied).	1 (very dissatisfied) to 10 (very satisfied)
**How satisfied were you with the online guidance (online feedback and other contact) that you received during i-Sleep?**<br>Rate your satisfaction on a scale of 1 (very dissatisfied) to 10 (very satisfied).	1 (very dissatisfied) to 10 (very satisfied)
**To what extent were the following advice and techniques from the online i-Sleep module helpful for reducing your sleep problems?**<br>Rate on a scale from 1 (not at all effective) to 10 (highly effective):	
Adjusting lifestyle (alcohol, exercise, relaxation during the day, etc.)	Scale: 1 (not at all effective) to 10 (highly effective)
Adjusting your bedroom environment (light, sound, comfort, etc.)	
Gradually winding down your day (fixed evening ritual)	
Applying sleep restriction (limiting time spent in bed)	
Maintaining a fixed day-night rhythm (consistent bedtimes)	
Implementing 15-minute "worry time" (evaluate the day)	
Implementing other rumination exercises (blocking thoughts, etc.)	
Performing relaxation exercises (muscle relaxation, etc.)	
Adjusting incorrect, unhelpful thoughts about sleep and insomnia (formulating alternatives)	
Creating a plan for the future (evaluation, continuing learned techniques)	
**Do you have suggestions for improving the online i-Sleep module and/or its personal guidance?**	( ) Yes, namely ...<br>( ) No
**If Yes:** What are your suggestions for improving the online i-Sleep module and/or its personal guidance?	Open text field (optional)

2.2. At T2 (8-month follow-up): long-term adherence

Item	Response option
**To what extent have you carried out the following activities since you followed/completed the online sleep module?**<br>Rate on a scale from 1 (never performed again) to 10 (performed daily):	
Reflecting on your lifestyle and making necessary changes (alcohol use, exercise, daytime relaxation, etc.)	Scale: 1 (never performed again) to 10 (performed daily)
Reflecting on your bedroom environment and making necessary changes (light, noise, comfort, etc.)	
Gradually winding down the day with a fixed evening routine	

Limiting the time spent in bed to increase sleep pressure	
Maintaining fixed bedtimes and wake-up times	
Performing rumination exercises to reduce ruminative thoughts (15-minute worry time, etc.)	
Performing relaxation exercises for sufficient relaxation during the day	
Reflecting on incorrect thoughts about sleep and insomnia, and formulating alternative thoughts	
Referring to and following the plan you created for the future	
**Did you engage in any other activities to reduce your sleep problems after completing the online sleep module?**	( ) Yes, namely ...<br>( ) No
**If Yes:** What other activities did you do to reduce your sleep problems after completing the online sleep module?	Open text field (optional)
**Do you have any further comments or suggestions?**	Open text field (optional)

3. Baseline assessments

BPDSI

BPD symptom severity was assessed with the semi-structured clinical interview Borderline Personality Disorder Severity Index-5 4-week-version (BPDSI-5-4wk) [1], administered in person or via videocall. The BPDSI-5-4wk measures symptom severity of the past 4 weeks and consists of 70 items divided over nine subscales corresponding to the DSM-5 BPD criteria: abandonment, relationships, self-image, impulsivity, (para)suicidal behavior, mood swings, feelings of emptiness, anger attacks, and dissociation and paranoid ideation. Items are scored on a scale from 0 (never) to 10 (daily), except for the subscale ‘self-image’, which is scored from 0 (completely absent) to 4 (clearly and predominantly present) and multiplied by 2.5 to obtain the mean subscale score. The BPDSI-5-4wk demonstrated excellent internal consistency, good interrater- and test-retest reliability, and good construct- and criterion validity [1].

ISI

Insomnia severity was measured with the ISI. The ISI is a self-report questionnaire consisting of 7 items rated on a scale from 0 to 4, with higher sum scores indicating more severe insomnia [2]. The ISI has adequate internal consistency, is sensitive to change, and has been validated for online use [2, 3].

SCID-5-PD

The SCID-5-PD was used to diagnose PDs according to the DSM-5 criteria [4]. It consists of 119 items that can be scored either as absent (0), subthreshold (1) or above threshold (2). To shorten the time of administration, prior to administering, patients fill out the SCID-5 self-report personality questionnaire (SCID-5-SPQ) consisting of 106 yes/no questions as a screening tool. Only the sections that scored above threshold (in addition to the BPD section) were administered during the SCID-5-PD interview. The SCID-5-PD is a revision of the SCID-II, which in Dutch samples proved to be a reliable and valid instrument, with good test-retest reliability [5] and interrater agreement for each PD (BPD: $\kappa = .91$) [6].

MINI

Possible other comorbid psychiatric disorders were assessed with the Mini-International Neuropsychiatric Interview (MINI) [7]. For this study, the sections concerning MDD, GAD, SAD, PD, and PTSD were administered.

4. Codebook

Theme	*Code*	Description / examples
**Reach**		
Reasons for participation / expectations	Long-standing sleep problems	• Participant has had sleep problems for a long time.<br>• Experiences many sleep-related complaints.
	Hope for symptom reduction	• Participant hopes that participating in the study will help them to sleep well or better.
	Scientific contribution	• Motivation to participate in the study stemmed from wanting to contribute to scientific research.
	Participation on advice from therapist	• Advice from a mental healthcare therapist was a reason for participating in the study.
	Influence of i-Sleep in context of an RCT	• Hesitation to participate due to randomization.<br>• Motivation to participate and adhere to i-Sleep was driven by a sense of responsibility towards the researcher.<br>• The participant was unclear that the study was scientific research and what that entailed.
**Effectiveness**		
Perceived effectiveness	Effect on BPD symptoms	• Participant experienced a positive effect of i-Sleep on BPD symptoms, e.g.:<br>o Less restlessness<br>o Less impulsive behavior (e.g., alcohol use, 'hanging out,' risky behavior) during initial sleep restriction phase: fatigue and maintaining fixed bedtimes<br>o Less worrying<br>• Participant experienced no effect of i-Sleep on BPD symptoms.<br>• BPD symptoms did decrease, but the participant did not feel this was due to better sleep.<br>• Participant experienced more fatigue/mood issues at the start of sleep restriction.
	Effect on sleep / insomnia symptoms	• Participant experienced reduced sleep problems due to i-Sleep, e.g.:<br>o Better sleep quality, longer sleep duration, more consistent routine / less variable bedtimes, shorter sleep onset latency, less wakefulness during the night, does not wake up earlier than desired.<br>• Participant experienced more daytime fatigue (as a result of sleep restriction).
	Positive effect on lifestyle	• Participant experienced a positive effect of i-Sleep on eating habits.
**Implementation**		
Session 1: sleep hygiene	*Winding down the day is helpful*	• Winding down the day through an evening routine helped the participant reduce their sleep complaints.<br>• Winding down the day helped with relaxing more.

		• Winding down the day helped with dwelling less on "nasty things," "annoying information," "the turmoil."
	Lifestyle adjustments are helpful	• Taking more moments of rest and relaxation during the day was helpful. This led to less worrying in the evening.<br>• Exercising and being more physically active was helpful.<br>• Drinking less alcohol before bed was helpful.
	Helpfulness of adjusting bedroom surroundings	• Adjusting the bedroom surroundings was helpful. Realizing the importance of being comfortable in bed.<br>• Adjusting the bedroom surroundings was not possible, for example, due to factors beyond one's control or practical reasons.<br>• Changes to bedroom-related sleep hygiene were not necessary.
Session 2: sleep restriction and fixed bedtimes	*Very difficult, but effective*	• The participant found sleep restriction very difficult, but at the same time, it was very effective for improving sleep.<br>• Sleep restriction was very demanding to implement alongside other complaints.<br>• Sustaining sleep restriction and fixed bedtimes was difficult on days off and weekends due to a lack of morning obligations or evening activities.
	Adhering to sleep restriction and fixed bedtimes is helpful	• Sleep restriction and adhering to fixed bedtimes was helpful.<br>• Participant was surprised by how much sleep restriction and fixed bedtimes helped.<br>• Sleep restriction had an immediate effect.<br>• Was considered the most effective component of i-Sleep.<br>• Sleep restriction was helpful, but only moderately effective.<br>• Helped by creating more evening fatigue.<br>• Helped by creating more regularity in wake-up time.
	Concrete / clear exercise	• Sleep restriction and adjusting bedtimes were concrete exercises to apply.
	Sleep restriction was not applied	• Did not manage to apply sleep restriction due to rotating shifts.
Session 3: worrying en relaxation	*Worry time was not helpful / not applied*	• 15-minute worry time did not work well; it was not enough for the severity of the worrying/negative thoughts.<br>• Worry exercises (worry time and thought-blocking techniques) were not applied.<br>• Worry exercises were already known from long-term (BPD) symptoms and were not used as they had proved unhelpful in the past.<br>• Did not manage to find a moment for worry time; the participant didn't think of it.

	Audio relaxation exercises were not helpful	• Relaxation exercises with audio fragments (focused on bodily relaxation and breathing) were not helpful and were therefore not used much.<br>• Felt uncomfortable, caused more tension, not relaxing.
	Audio relaxation exercises were helpful	• Relaxation exercises using audio were helpful.
	Moments of relaxation were helpful	• Integrating moments of relaxation during the day was helpful.<br>• Relaxation through activities (e.g., creative, walking) was particularly effective.
Session 4: reframing negative thoughts about sleep	*Reframing negative thoughts was too difficult*	• Participant found reframing negative thoughts (too) difficult.<br>• Participant has many / only negative thoughts, struggles with their own thoughts, and cannot manage to change them to positive ones.<br>• Could not manage to come up with positive thoughts.
	Negative thoughts are not about sleep	• Participant has few/no negative thoughts about sleep.<br>• Negative thoughts are mainly about other things than sleep.
	Reframing negative thoughts was not helpful	• Participant found reframing negative thoughts / session 4 not helpful.
	Reframing negative thoughts was helpful	• Participant found reframing negative thoughts helpful.<br>• It was difficult but gradually got a little better at reframing negative thoughts about sleep.
Session 5: summary and future plan	*Review was helpful to not forget*	• Reviewing the content of the i-Sleep module was helpful to recall what had been discussed.
Digital module	*Use of digital platform – positive*	• Participant found the website easy to use; the i-Sleep sessions were easy to complete.<br>• Participant found the sleep diary easy to fill out.<br>• Participant found filling out the sleep diary helpful.
	Use of digital platform – negative	• Participant found it impractical that completed sessions could not be reviewed afterward.<br>• Being able to review one's own answers in the sessions was mentioned as a suggestion for improvement for the i-Sleep module.<br>• Participant often forgot the password for the website account.
	Positive experiences with online module	• The online module is accessible.<br>• The online module is logistically convenient. Easier to schedule alongside work, takes less time, no need to travel to a location.<br>• Information is easier to remember than in a face-to-face appointment.<br>• It was nice that it could be done at one's own pace.

		• Having to fill out and keep track of the online sessions oneself created a greater sense of responsibility and encouraged active engagement, a 'doing' attitude.
	Negative experiences with online module	• Participant dislikes video calls; feels uncomfortable during video calls; is uncomfortable seeing themselves.
	Preference for online vs. non-online	• Participant prefers an online treatment.<br>• Participant prefers a combination of online and face-to-face treatment.
Therapist guidance	*Added value of written feedback*	• Participant found the written feedback helpful.<br>• Helps with remembering the content better.<br>• Knowing that someone is reading along.<br>• Helps with getting extra tips; thinking along with individual adjustments.
	Written feedback was necessary	• Participant found the written feedback necessary for following and adhering to i-Sleep.<br>• Provides motivation.<br>• Knowing that someone is reading along and cares about you gives a sense of support.
	Written feedback was not necessary	• Participant found the written feedback not necessary for following i-Sleep.<br>• The content of the i-Sleep sessions was clear and comprehensive enough.<br>• The feedback was not a nuisance, but not needed.
	Added value of video call appointments	• Participant found the video call appointments helpful.<br>• Video call appointments are important due to personal contact. Contributes to a sense of personal contact; the idea that a 'real' person is behind it. Without the video calls, an online treatment feels more distant.<br>• Video call appointments ensure continuous contact. Extra contact with a therapist was helpful.<br>• Video call appointments were helpful for reviewing the content of the sessions and reflecting on how things went with the exercises and behavioral adjustments.
	Video call appointments were necessary	• Participant found the video call appointments necessary for following and adhering to i-Sleep.<br>• Contact only via email is not enough.
	Frequency of guidance	• Participant found the frequency of the written feedback sufficient.<br>• The guidance was not too much but provided enough support.<br>• No changes desired in the amount of written feedback and video call guidance.<br>• Preference for more video call appointments.

	Necessity of reminders from the therapist	• Receiving reminders from the therapist was helpful and necessary.<br>• Phone reminders were necessary to not forget.
	Experiences with therapist contact were positive	• Participant had a positive experience with the therapist contact.<br>• Contact with the therapist provided a sense of calm.<br>• Contact with the therapist was not coercive.<br>• Therapist was easily accessible and flexible in scheduling the video call appointments.
Combination of sleep and BPD treatment	*Timing of i-Sleep relative to BPD treatment*	• Experiences of the participant regarding the timing of i-Sleep relative to upcoming BPD treatment.<br>• Difficult to make a statement about the optimal timing.<br>• Nice to follow the sleep treatment during the waiting period for BPD treatment. To already work on (lighter) issues before starting BPD treatment.<br>• Preference for following sleep treatment prior to BPD treatment.<br>• Sleep treatment should not only be given after BPD treatment.<br>• Participant liked following sleep and BPD treatment simultaneously.<br>• Participant found it to be a lot to follow sleep and BPD treatment simultaneously.<br>• The preference for simultaneously following sleep/BPD treatment depends on how much time you have, and motivation is also important.
	Necessity of specific attention for sleep	• Participant states that sleep treatment should be a part of BPD treatment.<br>• Participant found it helpful to be specifically engaged with sleep. Separate attention for treating sleep problems is necessary.<br>• It is necessary for sleep treatment to return in subsequent (BPD) treatment. To (better) sustain what was learned in i-Sleep, it would have been necessary for this to return in a subsequent treatment.
	Advantages of integrating sleep + BPD treatment	• Participant would prefer not to have separate therapists / locations for sleep and BPD treatment.<br>• Participant found it inconvenient to have to work in multiple systems (e.g., website / app / workbook).
**Maintenance**		
Continued and future use	*Sustained application of techniques*	• Participant has continued to apply i-Sleep techniques and wants to continue applying them in the future, e.g.:<br>○ Rest, moments of relaxation, and relaxing activities during the day.<br>○ Winding down the day.<br>○ Sleep restriction and adhering to fixed bedtimes.
	Use of i-Sleep techniques is no longer necessary	• Participant no longer applies i-Sleep techniques because sleep is now good; no longer needed.

Planning to continue applying in the future	• Participant plans to use i-Sleep again in the future if needed.
Relapse	• Participant has relapsed into old behavior or applied techniques have faded.<br>• After the i-Sleep sessions, everything comes to a complete halt.
Would recommend i-Sleep to others	• Participant would recommend i-Sleep to others.<br>• It is useful to be actively engaged with sleep.<br>• There are enough different tips, so there is something in it for everyone.

Supplementary results

Table S1

Median and interquartile ranges of quantitative treatment evaluation data (Supplement to Table 4).

iCBT-I components	Treatment evaluation T1: Effectiveness				Treatment evaluation T2: Adherence			
	Interview subsample (*n* = 5)		Total sample (*n* = 19)		Interview subsample (*n* = 5)		Total sample (*n* = 21)	
	Mdn (IQR)	Range	Mdn (IQR)	Range	Mdn (IQR)	Range	Mdn (IQR)	Range
Session 1								
Lifestyle adjustments	7.00 (1.00)	3 – 10	7.00 (3.00)	3 – 10	6.00 (3.00)	1 – 7	6.00 (2.00)	1 – 10
Bedroom surroundings	6.50 (2.75)	1 – 9	6.00 (2.00)	1 – 10	6.00 (5.00)	2 – 10	6.00 (3.00)	1 – 10
Evening ritual	7.00 (1.00)	4 – 10	7.00 (2.75)	4 – 10	6.00 (2.00)	1 – 10	6.00 (4.00)	1 – 10
Session 2								
Fixed bedtimes	7.00 (1.00)	5 – 10	7.00 (2.25)	4 – 10	5.00 (1.00)	1 – 8	5.00 (2.25)	1 – 10
Sleep restriction	7.00 (0.00)	5 – 10	6.00 (2.00)	1 – 10	5.00 (2.00)	2 – 10	5.00 (4.25)	1 – 10
Session 3								
Relaxation	7.00 (2.75)	5 – 10	6.00 (3.00)	3 – 10	3.00 (6.00)	1 – 10	3.00 (4.00)	1 – 10
Rumination	6.00 (4.50)	1 – 10	6.25 (3.63)	1 – 10	2.00 (3.00)	1 – 5	2.00 (3.50)	1 – 10
Session 4								
Sleep-related cognitions	5.00 (4.00)	2 – 9	6.00 (3.00)	2 – 10	6.00 (1.00)	1 – 10	5.00 (5.50)	1 – 10
Session 5								
Future plan	8.50 (2.25)	3 – 9	6.00 (3.00)	3 – 9	3.50 (6.00)	1 – 10	4.00 (5.00)	1 – 10
Overall satisfaction iCBT-I	6.00 (3.00)	5 – 8	6.00 (1.50)	3 – 8				
Satisfaction online guidance	8.00 (2.00)	6 – 9	7.00 (3.00)	4 – 9				

Note. This table presents the same data as Table 4 in the main manuscript but displays medians (Mdn) and interquartile ranges (IQR) to better reflect the data distribution. Quantitative insights were assessed using a 10-point Likert scale aimed at perceived effectiveness at T1 (1 = not at all effective, 10 = highly effective) and long-term adherence after completing the intervention at T2 (1 = never performed again, and 10 = performed daily).

Supplementary references

1 van Trigt S, Mendoza Alvarez M, van der Zweerde T, De Picker L, van Straten A, Arntz A, et al. Psychometric properties of the 4-week version of the Borderline Personality Disorder Severity Index-5. Compr Psychiatry. 2025;143:152634.

2 Morin CM, Belleville G, Belanger L, Ivers H. The Insomnia Severity Index: psychometric indicators to detect insomnia cases and evaluate treatment response. Sleep. 2011;34(5):601–8.

3 Thorndike FP, Ritterband LM, Saylor DK, Magee JC, Gonder-Frederick LA, Morin CM. Validation of the insomnia severity index as a web-based measure. Behav Sleep Med. 2011;9(4):216–23.

4 First MB, Williams JBW, Smith Benjamin L, Spitzer RL. Structured Clinical Interview for DSM-5® Personality Disorders (SCID-5-PD): APA Publishing; 2016.

5 Weertman A, Arntz A, Dreessen L, van Velzen C, Vertommen S. Short-interval test-retest interrater reliability of the Dutch version of the Structured Clinical Interview for DSM-IV personality disorders (SCID-II). J Pers Disord. 2003;17(6):562–7.

6 Lobbestael J, Leurgans M, Arntz A. Inter-rater reliability of the Structured Clinical Interview for DSM-IV Axis I Disorders (SCID I) and Axis II Disorders (SCID II). Clin Psychol Psychother. 2011;18(1):75–9.

7 van Vliet IM, de Beurs E. Het Mini Internationaal Neuropsychiatrisch Interview (MINI). Een kort gestructureerd diagnostisch psychiatrisch interview voor DSM-IV- en ICD-10-stoornissen [The MINI-International Neuropsychiatric Interview. A brief structured diagnostic psychiatric interview for DSM-IV and ICD-10 psychiatric disorders]. Tijdschr Psychiatr. 2007;49(6):393–7.
